# Are Jurors Intuitive Statisticians? Bayesian Causal Reasoning in Legal Contexts

**DOI:** 10.3389/fpsyg.2020.519262

**Published:** 2021-02-05

**Authors:** Tamara Shengelia, David Lagnado

**Affiliations:** ^1^Department of Experimental Psychology, University College London, London, United Kingdom; ^2^Department of Experimental Psychology, University College London, London, United Kingdom

**Keywords:** Bayesian reasoning, causal inferences, intuitive judgment, probabilistic reasoning, jury decision making, causal Bayes nets, explaining away, zero-sum

## Abstract

In criminal trials, evidence often involves a degree of uncertainty and decision-making includes moving from the initial presumption of innocence to inference about guilt based on that evidence. The jurors’ ability to combine evidence and make accurate intuitive probabilistic judgments underpins this process. Previous research has shown that errors in probabilistic reasoning can be explained by a misalignment of the evidence presented with the intuitive causal models that people construct. This has been explored in abstract and context-free situations. However, less is known about how people interpret evidence in context-rich situations such as legal cases. The present study examined participants’ intuitive probabilistic reasoning in legal contexts and assessed how people’s causal models underlie the process of belief updating in the light of new evidence. The study assessed whether participants update beliefs in line with Bayesian norms and if errors in belief updating can be explained by the causal structures underpinning the evidence integration process. The study was based on a recent case in England where a couple was accused of intentionally harming their baby but was eventually exonerated because the child’s symptoms were found to be caused by a rare blood disorder. Participants were presented with a range of evidence, one piece at a time, including physical evidence and reports from experts. Participants made probability judgments about the abuse and disorder as causes of the child’s symptoms. Subjective probability judgments were compared against Bayesian norms. The causal models constructed by participants were also elicited. Results showed that overall participants revised their beliefs appropriately in the right direction based on evidence. However, this revision was done without exact Bayesian computation and errors were observed in estimating the weight of evidence. Errors in probabilistic judgments were partly accounted for, by differences in the causal models representing the evidence. Our findings suggest that understanding causal models that guide people’s judgments may help shed light on errors made in evidence integration and potentially identify ways to address accuracy in judgment.

## Introduction

Legal decision making often involves causal reasoning under uncertainty. Jurors who make decisions in criminal cases are tasked with dealing not only with inherent uncertainty of a myriad of facts but also with disentangling the complexity of causal relations. For example, criminal law draws a distinction between factual and legal causes (Wilson, 2017). Factual causes focus on acts or omissions that have contributed to a harmful outcome while legal causes relate to the accountability and imputability aspect of the crime in question. Difficulty in establishing factual causation is compounded by factors such as intervening causes, self-harm by the victim, intervention by third parties and medical conditions. Examples of causes in legal cases include motives, recklessness, negligence and diminished capacity, mens rea and possible effects may involve evidence and actus reus. Additionally, assumptions that underpin judgments in legal contexts are based on causal models that jurors build during the course of the case hearing as well as their pre-existing beliefs (Pennington and Hastie, 1986, 1992).

Many of these aspects of legal cases can be represented using Causal Bayesian Networks (CBN). CBNs (Pearl, 2000; Fenton et al., 2018) represent structured causal relations and inferences. They offer a systematic way to capture intuitive probabilistic judgments and measure their alignment with normative belief updating standards, including the qualitative direction of updating as well as numeric computations. CBNs allow us to capture prior beliefs, uncertainty associated with legal evidence and complexity of causal structures (Lagnado and Gerstenberg, 2017). Prior beliefs, causes and effects in a legal case can be represented with nodes in CBNs and uncertainty can be summarized in associated probability tables (Fenton et al., 2013).

### Causal Bayes Networks and Normative Causal Judgments

The present study draws on an existing body of literature, according to which probabilistic learning and reasoning approximates Bayesian principles (Chater et al., 2006; Chater and Oaksford, 2008). Rottman (2017) argues that human reasoning about causality can be appraised in terms of causal Bayesian Networks and that probabilistic Bayesian models act as normative standards for judgment. Normative judgments can be evaluated from a qualitative (updating in the right direction) and a quantitative (accurate numeric judgments) perspective. The causal theory of reasoning suggests that people’s judgments follow the qualitative causal reasoning norms that approximate Causal Bayesian Networks (Sloman and Lagnado, 2015; Rottman, 2017). However, people’s belief updating does not fit the exact Bayesian computations.

Peterson and Beach (1967), who coined the term “man as an intuitive statistician,” argue that statistically accurate reasoning provides a good approximation of human inference. They observe that people take into account relevant factors and update beliefs in the right direction. Rottman and Hastie (2014) show that people often make causal inferences in the right direction; that is qualitatively, judgments are aligned with Bayesian norms. This is supported by previous studies (Waldmann, 2000; Sloman, 2005; Sloman and Lagnado, 2005; Meder et al., 2008; Baetu and Baker, 2009). Evidence regarding the quantitative aspect of normative reasoning suggests that quantitative accuracy is not as close as qualitative correspondence. Many studies observe deviations from Bayesian quantitative standards, demonstrating more conservative judgments than warranted by evidence used in belief updating (Phillips and Edwards, 1966; Rottman and Hastie, 2014). Peterson and Beach (1967) also highlight conservative tendencies in belief updating and posit that intuitive judgments observed in real life often deviate from statistically accurate normative judgments, making reasoning less quantitatively optimal. One major deviation from Bayesian reasoning is base-rate neglect (Tversky and Kahneman, 1982). This occurs when information supplied about the prevalence of a phenomenon in question is ignored and probabilistic reasoning takes place without factoring in base rates. Koehler (1996) argues that base rates are unlikely to be ignored in contexts where information is represented in the form of frequencies, when base rates are implicitly learned, directly experienced or more diagnostic than prior beliefs. In rich real life contexts such as the courtroom, base rates might be ignored as people’s decisions are informed not only by information presented at the trial but also by their prior beliefs and these two might be very different. In cases where a party fails to substantiate a disputed base rate with supporting evidence, this might be treated as evidence against the claim. Overall, evidence suggests that decision making in legal contexts may rely more on prior beliefs than on base rates. Bayesian models account for such prior beliefs.

Research by Krynski and Tenenbaum (2007) shows that errors in probabilistic reasoning can be explained by a misalignment between the evidence presented and the intuitive causal models constructed by participants. They were able to reduce judgment errors such as base rate neglect when participants were presented with a causal structure and numeric estimates could be clearly mapped onto this structure. Participants’ computations were closer to Bayesian estimates. It should be noted that probability estimates still were not completely accurate and the main improvement was observed in the qualitative updating. This suggests that exploring the causal structures that underlie legal cases may help shed light on the belief updating process in legal contexts and any potential deviations from quantitative Bayesian reasoning.

### Interpreting Competing Causes: Explaining Away and Zero-Sum

One area of difficulty in quantitative updating concerns the interpretation of competing causes. When two independent causes can explain a common effect, observing that this effect is present, increases the probability of both causes. However, if one then receives evidence that one of the causes has occurred, the probability of the other cause decreases. This pattern of judgment is known as ‘explaining away’ (Pearl, 1988). It suggests that a positive association between each of the competing causes and an effect implies a negative association between the causes conditional on knowledge of the effect. For example, in a legal case of intentional harm, if abuse and a disorder are considered to be causes of a common symptom, when evidence provides support for the presence of abuse, at the same time perceived probability of the disorder should be decreased, i.e., the disorder has been explained away.

In explaining away situations people struggle with both qualitative and quantitative aspects of judgments (Rehder, 2014; Rottman and Hastie, 2014, 2016; but also see Liefgreen et al., 2018; Tesic et al., 2020). From the qualitative point of view, the direction of inference is sometimes inaccurate and from the quantitative perspective, updating is too conservative, leading to the underweighting of evidence.

Research by Rehder and Waldmann (2017) focused on errors associated with explaining away inferences in causal reasoning. They showed that people tend to be more accurate when they experience situations for which they are drawing causal inferences compared to situations that are simply described. Results suggest that adherence to normative causal reasoning depends on how causal models are presented, whether they are described or experienced directly.

Another bias that people exhibit when reasoning about competing causes is the zero-sum fallacy. Zero-sum reasoning broadly represents thinking where gains in one area take place at the expense of another’s losses. In the context of causal reasoning, this is represented by treating evidence in support of a given cause as evidence against an alternative cause. In a recent study by Pilditch et al. (2019), people displayed a zero-sum bias when interpreting competing causes. When evidence was equally predicted by two competing causes, it was treated as irrelevant and as a result, was disregarded.

A balanced evaluation of evidence in legal cases involves weighing up evidence against competing hypotheses. These hypotheses are often about the causes that lead to outcomes under examination. Making accurate inferences requires not only correct interpretation of the weight of evidence, but also being able to correctly identify the hypotheses against which evidence is tested. Hypotheses can be considered mutually exclusive and exhaustive only when one (and only one) of the hypotheses can be true, ruling out any other explanation. For example, someone either dies from natural or unnatural causes. However, evidence in reality rarely warrants exclusivity and exhaustiveness of causes. There are usually many unknown possible causes of any piece of evidence. Being able to differentiate hypotheses that are not mutually exclusive and exhaustive is critical to avoiding the zero-sum fallacy, which occurs when hypotheses that are not mutually exclusive and exhaustive are erroneously treated as such.

### Diagnostic and Predictive Causal Reasoning

Inferences from causes to effects represent predictive reasoning and moving from effects to causes corresponds to diagnostic reasoning. In a study of diagnostic causal reasoning with verbal probabilistic expressions, such as “frequently,” “rarely,” “likely” and “probably,” Meder and Mayrhofer (2017) found that inferences based on qualitative verbal terms, which are more widely used in everyday life to express uncertainty than numerical expressions, match those that are drawn from numerical information only. Overall, the study provided support for the human ability to make accurate probabilistic judgments, closely aligned with normative standards of Bayesian causal reasoning.

Diagnostic reasoning is underpinned not only by probabilistic judgments about cause given effect, but also by causal relations that connect causes to effects (Meder et al., 2014). The plausibility of causal models, in particular, is seen as one of the key factors impacting diagnostic judgments. According to this study, errors in observed diagnostic inferences can often be explained by variations in underlying causal models.

Hayes et al. (2018) suggested that the role of causal models in normative judgments merits further study. The authors were interested in assessing whether representations of causal models facilitate Bayesian probabilistic judgments in terms of normative accuracy as well as reduction in error magnitude. Participants were provided with causal explanations for statistical information (e.g., false positives) and their judgments for the likelihood of the corresponding events were compared with normative standards. The study results suggest that while providing causal explanations does not result in improved normative judgments, it can still help alter people’s causal models by drawing attention to the statistical information which gets incorporated into causal structures.

### Are Jurors Intuitive Bayesian Statisticians?

While the normative interpretation of the Bayesian formula implies that beliefs about guilt will be updated based on evidence, the judgments may not always match the quantitative Bayesian norms even when the qualitative interpretation is accurate.

Previous research suggests that jurors are competent at evaluating scientific evidence but tend to show systematic errors in processing quantitative evidence under certain circumstances (Hans et al., 2011). The discrepancy between the observed and quantitative normative updating judgments increases with the amount of evidence (Schum, 1966). One cause for this discrepancy may be the increased difficulty of estimating the diagnosticity of the available evidence when it is expressed in high numerical values. Dartnall and Goodman-Delahunty (2006) claimed that people are not sensitive to the probative weight of the probabilistic evidence. Such empirical evidence prompted researchers to see jurors as incompetent in intuitive probabilistic reasoning, prone to errors and systematic violation of rational belief updating principles (Arkes and Mellers, 2002).

Thompson et al. (2013) criticize the claims that people are always conservative Bayesian thinkers; instead they provide evidence that belief updating in relation to the quantitative evidence in criminal cases is in line with Bayesian norms. The authors argue that methodological limitations in earlier studies may have resulted in inferring that deviations from Bayesian norms in participants’ observed judgments were more conservative than they actually were. Drawing on measures that were designed to address methodological shortcomings of previous studies, Thompson et al. (2013) found that while people at times engage in erroneous statistical reasoning, this is not always the case and people often reason in line with Bayesian belief updating models.

### Present Study

The present study explores how people update beliefs in light of evidence, examining alignment with Bayesian norms from a qualitative (direction of updating) as well as a quantitative (numeric computations) perspective. The study focuses on the following aspects of causal reasoning: (1) predictive inferences from effects to causes; (2) diagnostic inferences from causes to effects; and (3) explaining away inferences with competing independent causes.

The present study is based on a summary of a real case where a couple was accused of intentionally harming their baby. In this case, a young child was brought to hospital by his parents because they noticed the child had bleeding in his mouth. The parents had no explanation for the bleeding, and said that the child had not been involved in an accident. In our experiment participants are given two possible causes for the bleeding: abuse and a rare blood disorder.

Participants are provided with information about the hospital admission rates for children with this symptom for cases of abuse and rare blood disorder. The story mentioned that figures from previous hospital admissions suggest that 1 in 100 children admitted with bleeding to the mouth have been abused by their parents, and 1 in 1,000 have the rare blood disorder.

After presenting background information, further evidence was presented one piece at a time. This involved information about:

(1) Doctors noticing bruising on the child
(2) The hospital radiologist carrying out an X-ray on the child and reporting that the X-ray showed fractures.
(3) The child being tested for the blood disorder and testing positive.
(4) An independent expert radiologist employed by the prosecution re-examining the X-ray results and claiming there were no fractures.

**FIGURE 1:**
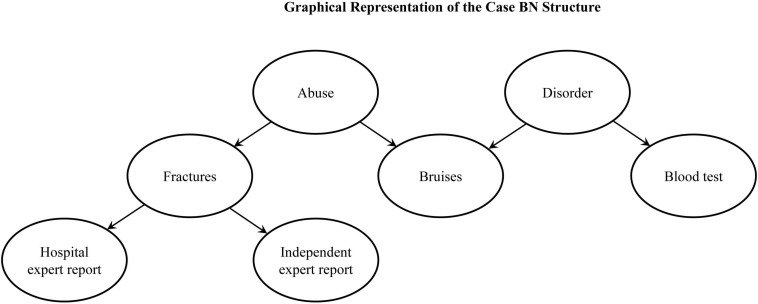
The graph represents the underlying Bayesian Network (BN) causal structure of symptoms and evidence in Experiments 1 and 2.

The causal structure of the case is presented in Figure 1.

## Experiment 1

The main goal of Experiment 1 was to examine whether participants’ beliefs are updated in line with Bayesian norms when dealing with competing causes (abuse and blood disorder) in a sequential inference task. Evidence was presented in stages, one piece of evidence at a time.

### Method

#### Participants

155 participants were recruited through Amazon Mechanical Turk to take part in the study. In all experiments, participation was restricted to respondents who had at least a 95% approval rating for their previous MTurk work. Participants were English speakers and based in the United States. Participants who were unable to correctly answer the comprehension check questions regarding the underlying causal structure, were excluded from the analysis, leaving 127 participants (49 female). The mean age was 33.9 (SD = 11.04, range 73–13 = 55). Out of the 127 participants included in the study, 36.2% had an undergraduate degree, 10% – Masters or Ph.D. degree, 32.3% completed a college education and 22% had no qualification.

#### Design and Procedure

In the present study base rates are presented in a frequency format as research (Hoffrage et al., 2015; Woike et al., 2017; Weber et al., 2018) suggests that natural frequencies are preferred over probabilities in Bayesian reasoning tasks to minimize errors in inferences. When presented with background information about the case, participants were told that “Figures from previous hospital admissions suggest that 1 in 100 children admitted with bleeding to the mouth have been abused by their parents, and 1 in 1,000 have the rare blood disorder.”

The following evidence was presented in stages: bruises, a hospital x-ray expert’s report, blood test results and an independent x-ray expert’s report. To examine the possibility of zero-sum reasoning when assessing evidence (Pilditch et al., 2019) we varied the instructions given to participants about the exclusivity and exhaustiveness of the causes (abuse and blood disorder).

Participants were divided into three groups according to the presentation format for the abuse and causes. The experiment consisted of the following conditions:

Condition 1: Abuse and disorder were presented as non-exclusive causes of the child’s bruises and bleeding.

Condition 2: Abuse and disorder were presented as non-exclusive and non-exhaustive causes of the child’s bruises and bleeding.

Condition 3: Control condition contained no statement about the relationship between abuse and disorder as causes of the child’s bruises and bleeding.

The dependent measures included the probabilistic judgments about the abuse and disorder as causes of the child’s symptoms. The probability judgments were recorded after introducing the background information as well as after exposure to each new element of evidence (bruising, a hospital radiologist’s report, blood test results and an independent radiologist’s report).

Information presented to participants specified that bruising was a common consequence of abuse and also of the blood disorder. It further stated that fractures were a common consequence of abuse, but not of the blood disorder.

Conditional probabilities elicited from the participants at the end of the experiment were used to construct their models of evidence evaluation based on Bayesian reasoning. Individual Bayesian network models were constructed for each participant. Actual probability judgments were compared to those predicted by these models. The differences between the probabilistic judgments predicted by the subjective models in line with Bayesian norms and the actual probabilistic judgments formed a dependent measure in this experiment. Probability judgments were compared to the individual causal models inferred from conditional probabilities. Subjective priors were compared to base rates supplied in the introduction.

The probability judgments in the experiment were recorded on a scale of 0 to 100%. Participants used an on-screen slider with numerical values to indicate their answer. Questions about the probability judgments used the following format: “What are the chances of …?”. On the slider response scale, 0% was labeled as “Very unlikely” and 100% as “Very likely”.

The magnitude of updating from one stage of evidence to another was calculated as a difference between the probability estimates at the present and previous evidence stage, at Stage 2 (Bruises), Stage 3 (Hospital expert report), Stage 4 (Blood test results), and Stage 5 (Independent expert report).

The experiment was hosted on Qualtrics^1^. Participants were given a legal case (see Table 1). After reading the background information which contained priors for the probability of abuse and disorder as possible causes for the child’s symptoms, participants were presented with four pieces of evidence in stages, one piece at a time. Starting from the introduction of the case background, participants were asked to provide their probability estimates for the abuse and the disorder as possible causes separately (“What are the chances that the parents abused the child?,” “What are the chances that the child has the blood disorder?”). This process was followed throughout the experiment, eliciting subjective probabilities for abuse and disorder every time new evidence was presented. The order of questions was fixed and followed the sequence of evidence presentation. Conditional probabilities were also elicited after all pieces of evidence were presented and included questions such as “If the child has the blood disorder, how likely is he to test positive?,” “If the child has fractures, how likely is the hospital radiologist to report that he has fractures?”). The procedure was adopted to track belief revision alongside the introduction of new evidence.

**TABLE 1:** Task formulation.

**In Experiments 1 and 2**	**Text**	**Responses**
Introduction	A young child was brought to hospital by his parents because they noticed the child had bleeding in his mouth. The parents had no explanation for the bleeding, and said the child had not been involved in an accident. Doctors suggested two possible causes for the bleeding: abuse and a rare blood disorder.	
Statistical information	Figures from previous hospital admissions suggest that 1 in 100 children admitted with bleeding to the mouth have been abused by their parents, and 1 in 1000 have the rare blood disorder.	When responding to questions about base rates, this information remained visible to participants.
Questions after introduction and each stage of evidence presentation	• What are the chances that the parents abused the child? • What are the chances that the child has the blood disorder?	• Responses on a scale of 0% to 100%
Conditional probability questions showing probability of an event given the occurrence of other event(s)	**Questions about the bruises** • If the child has been abused but does NOT have the blood disorder, how likely is he to have bruises? • If the child has NOT been abused but does have the blood disorder, how likely is he to have bruises? • If the child has been abused and also has the blood disorder, how likely is he to have bruises? • If the child has NOT been abused and does NOT have the blood disorder, how likely is he to have bruises? **Questions about the blood test** • If the child has the blood disorder, how likely is he to test positive? • If the child does NOT have the blood disorder, how likely is he to test positive? **Questions about the fractures** • If the child has been abused, how likely is he to have fractures? • If the child has NOT been abused, how likely is he to have fractures? **Questions about the hospital radiologist report** • If the child has fractures, how likely is the hospital radiologist to report that he has fractures? • If the child does NOT have fractures, how likely is the hospital radiologist to report that he has fractures? **Questions about the independent radiologist report** • If the child has fractures, how likely is the expert radiologist to report that he has fractures? • If the child does NOT have fractures, how likely is the expert radiologist to report that he has fractures?	• Responses on a scale of 0 to 100 where “o” = Very unlikely, “100” = Very likely)

### Results

The effect of Evidence and Condition on the abuse probability judgments in the observed data was examined with a mixed ANOVA with Condition as a between-subject and Evidence Stage as a within-subject variable. Following a Greenhouse-Geisser correction, the main effect of evidence was statistically significant, *F*(2.545, 315.587) = 85.298, *p* < 0.001, and partial eta squared = 0.408. Pairwise comparisons indicated that there was a statistically significant shift in beliefs about abuse at each stage of evidence, suggesting that participants integrated evidence and revised beliefs following the presentation of evidence. There was no main effect of Condition, *F*(2, 124) = 1.201, *p* = 0.304.

A mixed ANOVA was carried out to explore the effect of Evidence Stage and Condition on the observed subjective probability judgments for disorder. Similar to the abuse probability judgments, a significant main effect of Evidence was found following a Greenhouse-Geisser correction, F(2.293, 284.339) = 479.268, *p* < 0.001, partial eta squared = 0.794. There was no effect of Condition, *F*(2, 124) = 0.127, *p* = 0.881. *Post hoc* comparisons using the Bonferroni test indicated no difference between prior beliefs (*M* = 0.269, SD = 0.021) and revised beliefs after evidence about bruises (*M* = 0.282, SD = 0.022). All other stages of belief updating, including the first expert’s report (*M* = 0.157, SD = 0.018), blood test results (*M* = 0.945, SD = 0.011) and the second expert’s report (*M* = 0.867, SD = 0.022) showed differences in beliefs compared to the previous stage. Subjective priors were considerably higher (*M*_*abuse*_ = 0.534, SD = 0.305; *M*_*disorder*_ = 0.267, SD = 0.238) than the objective priors (0.01 and 0.001, respectively) supplied as part of the case scenario.

**FIGURE 2:**
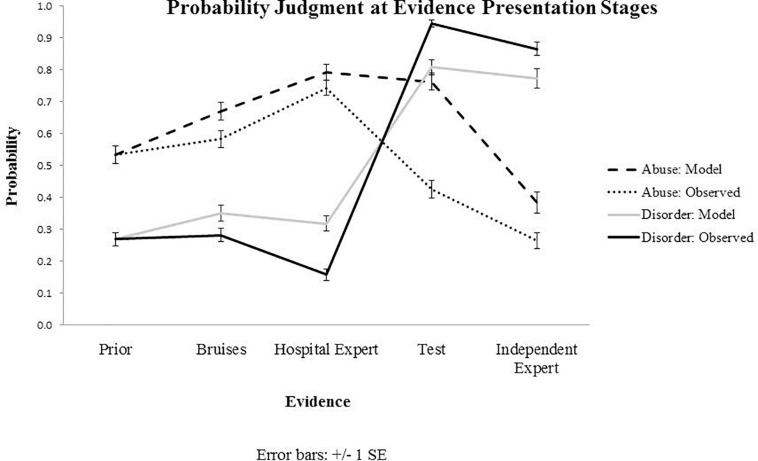
Results from Experiment 1: Observed and predicted (model) probability judgments at each evidence presentation stage, starting with prior beliefs and capturing belief updating following the evidence about bruises, hospital expert report, blood test results and independent expert report. Priors presented on the graph for both observed and predicted values represent subjective priors set by the participants.

**FIGURE 3:**
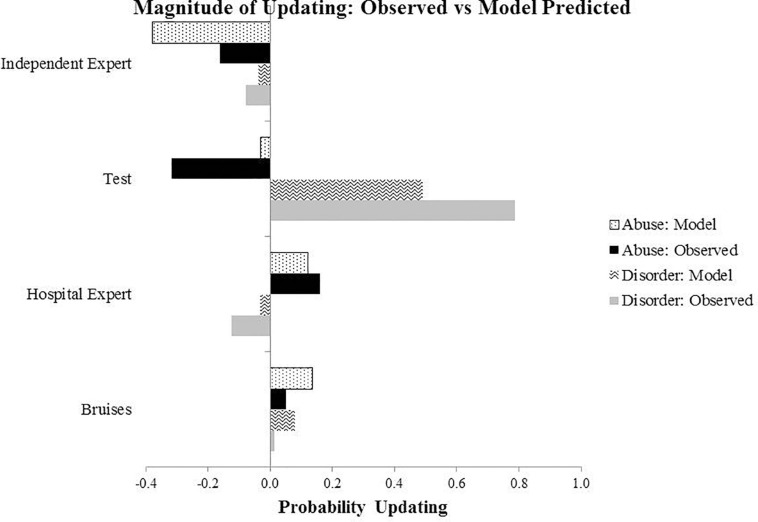
Results from Experiment 1: The graph shows extent of updating at four stages of evidence presentation, calculated as a difference between the probability estimates at the present and previous evidence stage. For example, for the Bruises stage, this is calculated by subtracting the probability value at the previous stage (prior elicitation) from the present stage (evidence of bruises).

Individual Bayesian belief updating models were obtained using thegRain package in R (Højsgaard, 2012). Differences between the observed and predicted (Bayesian) probability judgments during the belief updating process are summarized in Figures 2, 3, which draw on the participants’ own priors.

**FIGURE 4:**
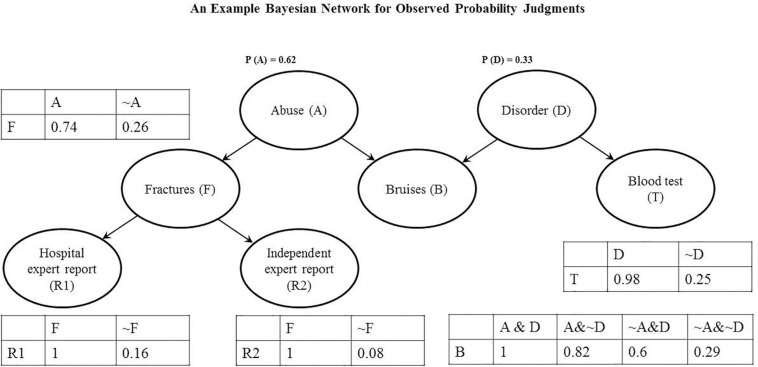
Individual Bayesian Network: The graph shows a Causal Bayesian Network and corresponding probability tables for one of the participants from Experiment 1.

An example Bayesian belief updating model is presented in Figure 4.

### Discussion

Differences between the observed and predicted judgments were found to be significant for all pieces of evidence, including bruising symptoms, hospital radiologist’s report and test results with regards to the abuse and disorder-related probability judgments indicating that the participants’ probabilistic judgments were different from exact Bayes computations. However, judgments were qualitatively in the right direction. There was no significant difference between conditions in belief updating, indicating that making the non-exclusivity and non-exhaustiveness of causes explicit did not affect probability judgments.

## Experiment 2

Experiment 2 focused on testing belief updating when participants were explicitly told, just before each probability judgment, that the causes in the study were non-exclusive and non-exhaustive. The purpose of this experiment was to determine the effect of bringing participants’ attention to the non-exclusivity and non-exhaustiveness of causes on the accuracy of judgments. This would allow us to rule out lack of understanding of the causal structure as a contributing factor to biased judgments observed in Experiment 1. The following statement was included at every stage of subjective probability elicitation: “Note that it is possible that both causes are true: e.g., that a child has been abused and has the disorder; it is also possible that neither are true, and that the symptoms arise due to other causes.” Additionally, participants’ understanding of the case causal structure was tested.

### Method

#### Participants

93 participants were recruited using the same protocol as in Experiment 1. As in Experiment 1, participation was restricted to respondents who had at least a 95% approval rating for their previous MTurk work. Participants were English speakers and based in the United States. The mean age was 35.08 (SD = 12.64, range 74–19 = 55). Out of the 93 participants (52 female) included in the study, 44% had an undergraduate degree, 21.5% – Masters or PhD degree and 23.7% completed a college education.

#### Design and Procedure

The procedure, instruction, and materials, including the questions were identical to those used in Experiment 1 except there was only one Condition, which corresponded to Condition 2 in Experiment 1. Additionally, at the end of the task we included questions to elicit participants’ causal models, focusing on the links included in the case model (Figure 1). Questions followed the format: “Did A cause B?”

### Results

A repeated measures ANOVA with a Greenhouse-Geisser correction showed that mean probability estimates differed significantly between the evidence presentation stages [*F*(2.631, 194.728) = 300.192, *p* < 0.001, partial eta squared = 0.802], observed and model judgments based on Bayesian predictions [*F*(1, 74) = 45.09, *p* < 0.001, partial eta squared = 0.379], but not between the abuse and disorder probability judgments [*F*(1, 74) = 1.334, *p* = 0.252].

Subjective priors were higher (M_*abuse*_ = 0.467, SD = 0.313; M_*disorder*_ = 0.441, SD = 0.325) than the objective priors (0.01 and 0.001, respectively) supplied as part of the case scenario.

**FIGURE 5:**
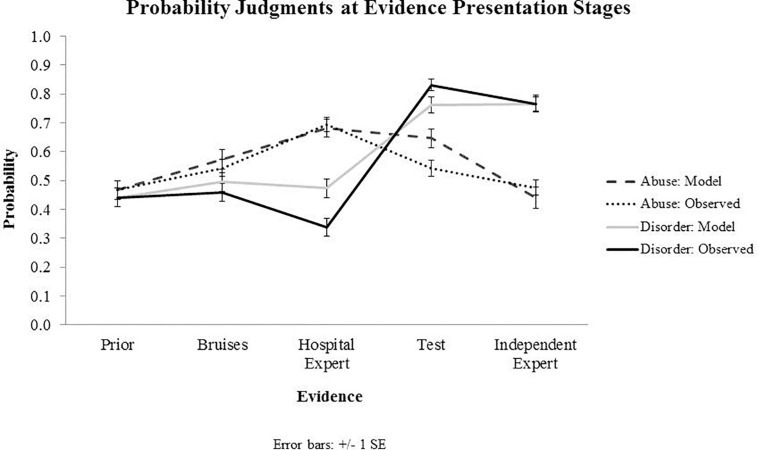
Results from Experiment 2: The graph presents observed and predicted (model) probability judgments for Abuse and Disorder at each sequential stage of evidence presentation. Priors presented on the graph for both observed and predicted values represent subjective priors set by the participants.

**FIGURE 6:**
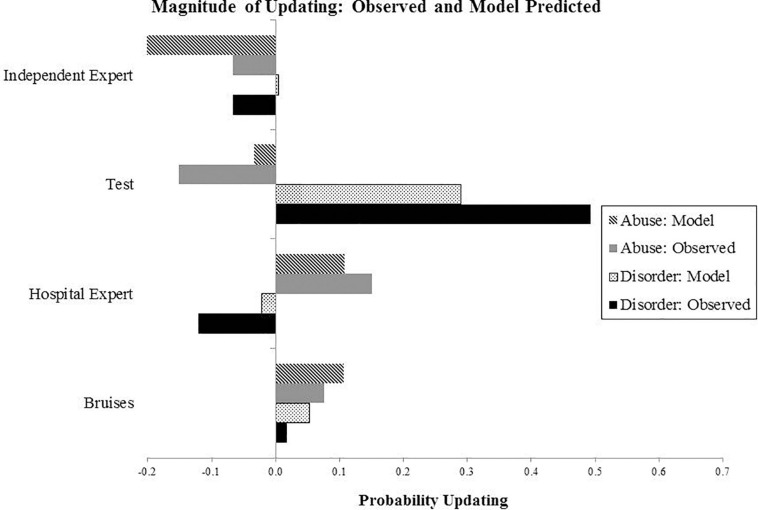
Results from Experiment 2: The graph shows extent of updating at four stages of evidence presentation, calculated as a difference between the probability estimates at the present and previous evidence stage. For example, for the Hospital Expert evidence stage, this is calculated by subtracting the probability value at the previous stage (evidence of bruises) from the present stage (evidence of the hospital expert report).

Belief updating, drawing on the participants’ own subjective priors, is summarized in Figures 5, 6.

To check for the general accuracy of the underlying causal models, we tested for the links that were included in the causal structure of the case model (Figure 1) as well as links that were incompatible with the model. Results for the links that were used in Experiment 1 to test comprehension of causal models as a basis for screening participants who did not answer the questions correctly, showed that the accuracy of responses was above the chance level. Participants correctly identified the causal structure. Specifically, participants were able to correctly identify that bruising was a common consequence of abuse and also of the blood disorder and that fractures were a common consequence of abuse, but not of the blood disorder. In response to questions whether a certain causal link was present or not, participants were able to correctly identify that Abuse could cause Bruises above the chance level, i.e., above 0.5 (0.53), Disorder could cause Bruises (0.78), and Abuse could cause Fractures (0.52). With regard to a causal link that was not part of the case causal structure such as Disorder causing Fractures, participants’ responses showed that participants were able to correctly exclude this link from their individual causal representations (0.27).

### Discussion

Participants updated beliefs in the direction predicted by normative Bayesian judgments on most occasions. However, as in Experiment 1, we found instances of under- and over-estimation of evidence in quantitative belief updating. This was particularly evident when integrating evidence that supported both Abuse and Disorder as possible causes (e.g., evidence of bruises), which led to the under-weighting of evidence and belief updating far lower than mandated by Bayesian normative judgments. Another instance of inaccurate quantitative judgment was following the positive blood test results, which showed that participants attributed excessive weight to evidence.

## General Discussion

The findings from both experiments suggest that in legal decision making people qualitatively update their beliefs in line with Bayesian norms. In our experiments, participants’ belief updating was qualitatively aligned with normative judgments, i.e., probability judgments increased or decreased in the same direction as predicted based on Bayesian norms. This was observed with both predictive inferences (e.g., increases in the probability of abuse increased the probability of fractures), diagnostic inferences (e.g., evidence of the hospital radiologist report raised the subjective probability of fractures) and explaining away inferences (e.g., evidence of positive test results raised the probability of the blood disorder and decreased the probability of abuse by explaining away). While most judgments fit with qualitative predictions of Bayesian models, an exception is observed at the final stage of evidence presentation where the subjective probability of blood disorder was lowered slightly rather than raised. This can be explained by exposure to conflicting expert reports, which may have decreased the perceived reliability of reports, resulting in a greater skepticism toward the blood test results.

Overall the results indicate that people’s qualitative reasoning is mostly accurate and follows qualitative predictions of Bayesian models in predictive, diagnostic and explaining away inferences. These findings reinforce results from previous studies where Bayesian probabilistic reasoning was observed (e.g., Thompson and Newman, 2015).

Results from both experiments indicate that people tend to ignore the priors provided as part of the background case information and set their own subjective priors. The subjective priors in both experiments were significantly higher than the objective priors offered in the case summary. Prior knowledge and expectations, underlying causal models may have contributed to setting higher priors than suggested by base rates.

Previous research about explaining away inferences is not conclusive (Morris and Larrick, 1995; Oppenheimer and Monin, 2009; Rehder, 2014; Rottman and Hastie, 2014, 2016; Tesic et al., 2020) and offers different views on challenges associated with explaining away inferences. Our experiments highlighted that people are able to navigate the explaining away type of scenarios and make accurate judgments about competing causes that fit with qualitative Bayesian predictions.

Prior evidence on people’s ability to make quantitative probabilistic judgments aligned with Bayesian norms is not definitive, with studies indicating either under- or over-estimation of evidence. In the existing body of literature, a unified mechanism for explaining an excessively low and high weight attributed to evidence has not been decisively established. Our findings show both types of departures from quantitative normative judgments: under-weighting of evidence when participants update beliefs based on the evidence of bruises and over-weighting of evidence following the evidence of the positive test result. Both these findings can be explained with zero-sum reasoning, which provides insights into how people integrate evidence when dealing with competing causes (Pilditch et al., 2019).

Zero-sum reasoning represents thinking whereby the gains of one person take place at the expense of another’s losses. A zero-sum model of the world presumes a finite and fixed amount of resources in the world, which necessitates a competition for these resources. In the context of competing causes, when two causes equally predict the same evidence, zero-sum thinking treats such evidence as neutral because it tacitly assumes that the causes are exclusive and exhaustive accounts of the evidence. For example, in our experiments the evidence of bruises which was predicted by both the abuse and disorder hypotheses, the evidence was treated as neutral, resulting in only slight increase in the probability of both, which was considerably lower than expected by Bayesian updating.

Zero-sum thinking also accounts for the over-weighting of evidence which was observed in the case of the excessive decrease in the probability of disorder following the hospital radiologist report and an excessive increase in the probability of disorder given the positive blood test result with a simultaneous disproportionate lowering of the probability of abuse. Excessive raising or lowering of probabilities points to the zero-sum nature of the reasoning involved in this process. Competing causes were perceived as exclusive, which had a hydraulic effect on the evidence interpretation: increasing the probability of one cause excessively decreased the probability of the other cause.

These findings are consistent with the results of Pilditch et al. (2019) who found that when interpreting evidence against competing causes, people treat evidence evaluation as a zero-sum game. The biased reasoning persisted even when the non-exhaustiveness of the hypotheses was made explicit. Our results also show that zero-sum thinking is observed despite the participants being made aware the non-exclusive and non-exhaustive nature of the competing causes.

Zero-sum reasoning in the context of our experiments suggests that under- and over-estimation of evidence are observed due to underlying assumptions about causes modeled on zero-sum principles. This type of reasoning may result in more accurate judgments when dealing with competing causes that are exclusive and exhaustive.

## Conclusion

Our study suggests that people are able to make qualitatively accurate causal inferences and update beliefs in the direction predicted by Bayesian norms. However, quantitative computations are not always accurate and show a gap between observed and normative judgments. Instances of underweighting and overweighting of evidence in our experiments can be explained by a zero-sum fallacy. This offers a useful perspective for shedding light on evidence integration in legal cases, where a balanced evaluation of evidence often involves weighing up of the evidence against competing hypotheses. These hypotheses are often about the causes that lead to outcomes under examination. Making accurate inferences requires not only correct interpretation of the weight of evidence, but also being able to identify the hypotheses against which evidence is tested. Being able to differentiate hypotheses that are not mutually exclusive and exhaustive is critical to avoiding a zero-sum fallacy.

## Data Availability Statement

The datasets generated for this study are available on request to the corresponding author.

## Ethics Statement

The studies involving human participants were reviewed and approved by Ethics Committee of the Department of Experimental Psychology, University College London. The patients/participants provided their written informed consent to participate in this study.

## Author Contributions

Both authors listed have made a substantial, direct and intellectual contribution to the work, and approved it for publication.

## Conflict of Interest

The authors declare that the research was conducted in the absence of any commercial or financial relationships that could be construed as a potential conflict of interest. The handling editor declared a shared affiliation, though no other collaboration, with the authors at the time of the review.

## Footnotes

1 www.qualtrics.com
